# Drug Development Papers in *PLoS Medicine*: How We Try to Spot a Winner

**DOI:** 10.1371/journal.pmed.0030547

**Published:** 2006-12-26

**Authors:** The PLoS Medicine Editors, Sanjeev Krishna

## Abstract

Deciding which drug development papers are appropriate for publication in a general medical journal is not simple; here, editorial board member, Sanjeev Krishna, and the editors discuss their strategy.

This month's *PLoS Medicine* publishes a paper describing the results of a very early drug discovery study. The nature of early drug development is to identify many promising compounds, only a small number of which eventually make it into routine treatment. Consequently, there are many more papers reporting promising leads in early drug development than ones that report efficacy in large clinical trials. Because of this natural attrition, medical journals (including this one) are often reluctant to publish intriguing results from the early stages of drug discovery. However, *PLoS Medicine* occasionally does publish such reports, and the manuscript by Dominique Mazier and colleagues [1] in this issue is an example. What made us and our advisers select this paper above the many similar ones we receive?

As editors, we ask several general questions about any submitted manuscript: how important is the research question (both globally and in relation to the journal's audience); what is the likelihood of the conclusions holding up over time (and when is it worth publishing preliminary results that would be important if confirmed but where confirmation is uncertain); and, for a highly selective general medical journal, do the results represent a substantial advance—be it in understanding pathogenesis, suggesting treatment options, or having implications for public health.

More specifically, for drug development papers, questions we consider include: how substantial is the disease burden that the drug would treat; are any treatments currently available (or do the available treatments have serious limitations); has a novel or interesting mechanism been developed; (or was a novel system used in the development of the drug); and finally, is there a realistic path to clinical development?

Malaria is clearly a topic of great relevance to *PLoS Medicine's* mission (of publishing papers on the diseases causing a high global burden of disease), and to our authors and readers. A substantial number of papers submitted to us concern malaria, and the 44 articles of all types on malaria that we have published have been cumulatively downloaded more than 100,000 times. The World Health Organization estimates that malaria is responsible for more than 1 million deaths per year, most of them young children living in Africa (see http://www.who.int/mediacentre/factsheets/fs094/en/). A recent *PLoS Medicine* paper by Mathers and Loncar [2] reported that malaria was the 12th leading cause of death worldwide in 2002. We also know there are substantial limitations of current treatments for malaria; resistance to antimalarial drugs is an increasing problem. Therefore, the development of a new drug, especially one targeting a stage of the parasite life cycle upon which very few other drugs act, is of great interest.

The paper by Mazier and colleagues describes the very early stages of such a development by identifying a novel compound against the liver stage of malarial parasites. Before Plasmodium spp. can cause the symptoms of malaria by multiplying in the bloodstream, the parasites must pass through the liver. Most current drugs target the blood stages. Hopefully, one day malarial disease might be prevented by an effective vaccine, but it will probably be many years before a vaccine that works by killing parasites at the liver stages of development is available. Another option for prevention would be drugs that act in the few days between a mosquito's inoculation of a patient with sporozoites and the time when parasites escape from the liver and develop into the blood stages of disease. In severe infections, such as those caused by P. falciparum, all parasites are released from liver cells after a few days, leaving none behind. But some Plasmodium species (*vivax* and *ovale*) can remain in liver cells for months, only re-emerging after the first wave of blood-stage parasites has produced fevers and thus eluding drugs inactive against liver stages. In the paper published in this issue, Carraz and colleagues applied a methodology for screening compounds active against the liver stages of P. falciparum and identified an inhibitor from aqueous extracts of Madagascan plants growing in eastern rain forests.

Few existing drugs kill liver stages of malarial parasites: two such drugs licensed at the moment are primaquine (an 8-aminoquinoline derivative) and the combination of atovaquone–proguanil. (Tafenoquine and bulaquine, structural relatives of primaquine, are not yet licensed.) A disadvantage of the 8-aminoquinolines is the toxicity caused by haemolysis of red blood cells in patients with some types of G6PD deficiency, a condition frequent in African populations.

In addition to the actual discovery, the technical aspects of this paper are worthy of note. One problem that has beset drug discovery in malaria is the difficulty of culturing the liver stage of the parasite in vitro. The effectiveness of the 8-aminoquinoline derivatives in killing liver stages was discovered well before the first cultures of blood stage parasites became possible in 1976. Since then, several other stages of development have been cultured in vitro, but culturing these stages is technically more challenging than the blood stages. Hence, the work of Mazier and colleagues can also be seen as a technical achievement, as their screening depended on a source of sporozoites and primary hepatocyte cultures established from liver biopsies. Antimalarial efficacy of one plant, Strychnopsis thouarsii, was initially assayed by experiments on P. yoelii (a rodent malaria infection) cultured in mouse hepatocytes. An extract with antimalarial activity in the lower end of the micromolar range was further analysed and structural analysis of the active chemical found a morphinan core that was chemically modified to give N-cyclopentyl (NCP)-tazopsine. This semi-synthetic derivative was shown to have a higher selectivity for hepatic parasites, compared with blood stages, and was also less toxic to host cells than the parent compound.

Further experiments showed that in vivo efficacy against sporozoite challenge with P. yoelii was confirmed and found to be best with NCP-tazopsine. Neither blood stages nor gametocytes were killed, confirming the unique hepatic selectivity of this class of antimalarials. One incidental but practical advantage of this selectivity of tazopsines, which is highlighted by the authors, is a diminished chance of selecting drug resistance because liver-stage parasites are much less numerous than blood-stage parasites.

But, even having acknowledged this study's merits, one might still ask whether it is reasonable for a general medical journal to devote limited publication resources to such an early-phase drug study. We would argue that, as failure to produce an effective drug is a common event, we should be prepared to accept a fair amount of uncertainty in such papers, especially those presenting technical advances, whether or not the particular drug in question succeeds.

And the compound that is the subject of this study has promise. Inhibitors of malaria not only have to cross multiple biological membranes to reach intra-hepatic parasites, but also have to elude metabolism and degradation by the very liver cells that host the parasite. Tazopsines have overcome some of these hurdles. The next stage of their development will be optimization of the chemically tractable lead inhibitor using screening for efficacy against liver stages of infection. Studies of tazopsines on P. vivax and P. ovale will also be of interest.

A truly selective drug would be a very welcome tool in the rather limited current armamentarium against Plasmodium parasites. Gambling publication resources on such an outcome seems a reasonable decision.
